# High-Sensitivity RT-LAMP for Molecular Detection of O’nyong-nyong (*Alphavirus onyong*)

**DOI:** 10.3390/pathogens13100892

**Published:** 2024-10-11

**Authors:** David Faísca-Silva, Gonçalo Seixas, Mónica Nunes, Ricardo Parreira

**Affiliations:** 1 Institute of Hygiene and Tropical Medicine (IHMT), NOVA University of Lisbon, Rua da Junqueira 100, 1349-008 Lisbon, Portugal; gseixas@ihmt.unl.pt (G.S.); ricardo@ihmt.unl.pt (R.P.); 2 Global Health and Tropical Medicine (GHTM), Rua da Junqueira 100, 1349-008 Lisbon, Portugal; 3 Departamento de Biologia Vegetal, Faculdade de Ciências, Universidade de Lisboa, Campo Grande, 1749-016 Lisboa, Portugal; msnunes@ciencias.ulisboa.pt; 4 3cE3c-Centre for Ecology, Evolution and Environmental Changes & CHANGE-Global Change and Sustainability Institute, Faculdade de Ciências, Universidade de Lisboa, 1749-016 Lisbon, Portugal; 5 Associate Lab in Translation and Innovation Towards Global Health, LA-REAL, Rua da Junqueira 100, 1349-008 Lisbon, Portugal

**Keywords:** arboviruses, isothermal amplification, loop-mediated isothermal amplification (LAMP), O’nyong-nyong, RT-LAMP assay

## Abstract

Mosquitoes serve as vectors for many arthropod-borne viruses (arboviruses) that are responsible for millions of human infections and thousands of deaths each year. Among these arboviruses, O’nyong-nyong virus (ONNV) is an African alphavirus mainly transmitted by *Anopheles* mosquitoes. ONNV can be detected through serological or molecular tests, the first showing cross-reactivity to co-circulating alphaviruses and requiring technically demanding confirmation, while the latter, usually based on real-time PCR, are costly and demand specific equipment. Isothermal amplification approaches, such as Loop-Mediated Isothermal Amplification (LAMP), should therefore provide a cost-effective, sensitive, and specific alternative for virus detection, suitable for the resource-limited regions where ONNV circulates up to the present time. Here, we describe the development and optimization of a rapid and highly sensitive (10 pfu/reaction) RT-LAMP assay for ONNV detection. Additionally, we demonstrate that it is possible to bypass the RNA extraction step, reducing sample handling time and costs. The final RT-LAMP_ONNV_ is a promising field detection tool for ONNV, enabling a better understanding of its impact and serving as a point-of-care diagnostic method.

## 1. Introduction

Vector-borne diseases are responsible for the deaths of millions of people each year [1]. In particular, mosquitoes are hematophagous arthropods with the heaviest direct and indirect impacts on human health, being the main vectors of well-known viruses such as dengue (DENV), Chikungunya (CHIKV), yellow fever (YFV), and Zika (ZIKV) [2,3]. These viruses are collectively known as arboviruses, which are biologically transmitted by systemically infected arthropods and represent an important public health issue [4,5].

Climate change, marked by rising global temperatures and shifting precipitation patterns, can impact vector development, pathogen replication, and breeding sites [6,7]. Combined with sociodemographic and economic factors, these environmental changes influence the distribution and frequency of disease vectors [8]. Consequently, climate change expands vector habitats [9], extending virus distribution from tropical regions to temperate areas like Europe [10], posing a global public health threat.

Arboviruses can cause from asymptomatic infections to those characterized by a wide range of symptoms, from mild to potentially life-threatening consequences, leading to considerable short- and long-term morbidity and mortality [11,12]. The most prevalent viruses affecting humans in such group are DENV, CHIKV, ZIKV, Japanese encephalitis (JEV), and West Nile (WNV) [3], with recent increases in Oropouche virus infections [13]. Less prevalent viruses include Mayaro (MAYV), Sindbis (SINV), Rift Valley (RFV), and O’nyong-nyong (*Alphavirus onyong*) (https://ictv.global/taxonomy/, accessed on 11 August 2024) [14]. O’nyong-nyong virus (ONNV) is a single-stranded positive-sense enveloped RNA virus belonging to the genus *Alphavirus*, family *Togaviridae* [15,16]. It was originally isolated in Uganda in 1959 during a major epidemic that affected at least 2 million people between 1959 and 1962 [17]. This mosquito-borne virus is the only arbovirus known to be primarily transmitted by *Anopheles* mosquitoes, particularly through the bite of *Anopheles funestus* and *Anopheles gambiae* [18]. ONNV is phylogenetically close to CHIKV, both belonging to the *Alphavirus* Semliki Forest antigen complex [15,17], and is typically detected through serological tests. Nevertheless, its genetic proximity with other co-circulating alphaviruses, especially CHIKV, often leads to cross-reactivity [19,20]. Currently, there are no reports of endemic cases from the WHO, CDC, or even local agencies, only sporadic imported cases to Europe [21], which suggests that the similarity to CHIKV could lead to a significant number of ONNV cases being misdiagnosed as CHIKV. Considering the limitations of serological tests, other methods, such as the plaque reduction neutralization test (PRNT), the gold standard for confirming virus identity, may be used to specifically identify ONNV, especially after presumptive serological identification [15]. However, ONNV detection cannot rely solely on PRNT due to several challenges, including the need of laboratory facilities equipped to handle viruses, dedicated equipment, qualified trained technicians, and adequate financial resources, which are often lacking in regions where ONNV circulates, particularly in Africa [15]. Molecular approaches like qRT-PCR (real-time/quantitative PCR preceded by reverse transcription), known for their sensitivity and specificity, could mitigate cross-reactivity issues [15] but require samples from acute infections collected during the viremic period and involve high costs and specialized equipment [15]. Thus, developing a more affordable alternative to qRT-PCR that maintains similar sensitivity and specificity is essential.

Isothermal amplification is a technical approach that supports nucleic acid amplification at a constant temperature with high specificity and sensitivity [22,23]. It encompasses several different possible techniques that do not require a temperature-changing system, making it suitable for use in regions with limited laboratory resources [24]. There are several isothermal amplification technologies, including Rolling Circle Amplification (RCA), Recombinase Polymerase Amplification (RPA), Strand Displacement Amplification (SDA), Helicase-Dependent Amplification (HDA), Nucleic Acid Sequence-Based Amplification (NASBA), and Loop-Mediated Isothermal Amplification (LAMP), among others [25]. LAMP is a technique that uses a set of four to six primers, giving rise to the characteristic LAMP dumbbell-shaped DNA [24]. Overall, this method stands out from others by combining the best characteristics of isothermal amplification for on-field diagnosis: it is fast, highly efficient, and its reaction temperature range ensures high specificity to the target [26]. Since the amplification is performed using a specific enzyme that has strong strand displacement activity—*Bst* DNA polymerase [27]—there is no necessity for a DNA denaturation phase. When the template is RNA, it is possible to perform RT-LAMP, like qRT-PCR. In this case, RT-LAMP is initiated by template reverse-transcription before a polymerase is added, or by using a modified version of a *Bst* DNA polymerase (*Bst* 3.0) that combines strand displacement and reverse transcriptase activities [28,29]. Additionally, RT-LAMP can be applied to non-pure samples bypassing template purification steps, resulting in a faster and more cost-effective methodology [30]. Consequently, an exponential highly sensitive and specific amplification occurs, making it an excellent alternative to RT-qPCR, particularly for point-of-care diagnosis in resource-limited settings [31].

This paper presents, for the first time, a simple, rapid, highly specific, and sensitive RT-LAMP assay (RT-LAMP_ONNV_) for ONNV detection using a *Bst* 3.0 polymerase directly in viral lysate samples. By eliminating complex sample processing steps and incorporating SYBR Green I for straightforward detection, this method represents a significant advance toward on-field diagnostic capabilities for ONNV.

## 2. Materials and Methods

### 2.1. Phylogenetic Analysis

The phylogenetic analysis of alphaviruses was conducted using two sequence datasets. The first included, when possible, two different complete genome accessions from each virus belonging to the *Alphavirus* genus. This dataset was created with randomly chosen (from NCBI Virus; https://www.ncbi.nlm.nih.gov/labs/virus/vssi/#/, accessed on 10 November 2023) representative sequences identifying the different viral genetic lineages, with varying dates of collection and diverse geographic origins. Two genomes from salmon pancreas disease virus (SPDV) were used as outgroups. A second phylogenetic analysis was conducted using an alternative sequence dataset including all available genomes of ONNV and 5 complete genomes of each CHIKV lineage. Multiple alignments of nucleotide (nt) sequences were performed for both datasets, using the iterative G-INS-1 method as implemented in MAFFT v7 [32], followed by trimming with GBlocks [33] to remove poorly aligned or divergent regions from the alignment, but making sure codon alignment was maintained. Phylogenetic reconstruction was carried out using the maximum likelihood (ML) optimization criteria as implemented in IQ-TREE [34]. For the alphavirus phylogenetic analysis (dataset 1), the best-fitting evolutionary model used was GTR + F + I + G4, as suggested by IQ-TREE. For the phylogenetic analysis exclusively for CHIKV and ONNV (dataset 2), the best-fitting evolutionary model used was GTR + I + F, again as indicated by IQ-TREE. The stability of the resulting ML trees was evaluated using both the classical bootstrap and SH-aLRT (Shimodaira–Hasegawa approximate likelihood ratio test), each conducted with 1000 resamplings of the original data. Bootstrap and aLRT values greater than 75% were considered significant. The phylogenetic trees were visualized using FigTree v1.4.3 software [35].

### 2.2. Cell Culture and Infection Dynamics

An O’nyong-nyong virus stock (UgMP 30/NR-51661, also known as the Gulu Strain) was obtained through BEI Resources-NIAID-NIH (https://www.beiresources.org, accessed on 19 October 2023). To examine the infection dynamics, Vero E6 cells (African green monkey epithelial cells) were seeded in Dulbecco’s minimum essential medium (DMEM) (Gibco^®^, Thermo Fisher Scientific, Waltham, MA, USA) supplemented with 10% fetal calf serum (FCS; Sigma-Aldrich, St. Louis, MO, USA), 2.5% HEPES (Sigma-Aldrich, St. Louis, MO, USA), 2% penicillin-streptomycin (Sigma-Aldrich, St. Louis, MO, USA), and 2% L-glutamine (Sigma-Aldrich, St. Louis, MO, USA), at 37 °C and 5% CO_2_ in humidified conditions. Monolayers at 90–95% confluency were infected with the virus at a multiplicity of infection (MOI) of 0.1 in a T25 culture flask (Corning^®^ Costar^®,^ Corning Incorporated, Corning, NY, USA). Adsorption was allowed to occur for 1 h and 30 min (with intermittent shaking), after which the monolayers were washed with sterile phosphate-buffered saline (PBS), followed by the addition of DMEM (Gibco^®^, Thermo Fisher Scientific, Waltham, MA, USA) cell culture medium supplemented with 2% FCS, 2.5% HEPES, 2% penicillin-streptomycin, and 2% L-glutamine. A mock infection was performed by replacing the viral inoculum with culture media. After infection, the cell morphology was periodically observed, and the cytopathic effects (CPEs) were registered at 0, 24, 48, 72, and 96 h post-infection (hpi) and compared to a mock infection (Supplementary Figure S1). A virus stock was obtained after one passage at 96 hpi, and the supernatant was stored in aliquots diluted 1:1 with calf serum, as suggested in the ATCC virology guide (available at https://www.atcc.org/resources/culture-guides/virology-culture-guide, accessed on 20 December 2023) for another arbovirus (Japanese encephalitis virus), at −80 °C until use for RNA extraction.

### 2.3. RNA Extraction

The total RNA was extracted from 150 µL of virus culture supernatant using a ZR Viral RNA kit™ (Zymo Research, Irvine, CA, USA), according to the manufacturer’s protocol. Viral RNA was eluted from the extraction column in a total volume of 15 µL, and the extracted RNA was kept at −80 °C until it was used.

### 2.4. RT-LAMP Primer Design

ONNV genome sequences were downloaded from the NCBI GenBank nt database (https://www.ncbi.nlm.nih.gov/genbank/, accessed on 23 December 2023). To maximize the *in silico* specificity of the ONNV amplification primers, all available complete ONNV genome sequences were compared with at least one sequence from each of the West African, East/Central/Southern African, and Asian lineages of CHIKV (given its phylogenetic and serological proximity to ONNV), as well as with SINV sequences, an alphavirus circulating extensively in the Old World. A multiple nt sequence alignment obtained with MAFFT vs. 7 (as mentioned above) was manually examined using BioEdit, identifying a region of interest (see Results for details) for primer design, the latter corresponding to one where the alignment of CHIKV/SINV was characterized by the presence of gaps when compared to the aligned ONNV genomes. To ensure that gaps were not artificially introduced by the alignment program, an alignment was also performed with amino acids (aas). RStudio (version 2023.12.1 + 402) was used to visualize the nt multiple sequence alignment detailed above. A plot from the MSA was drawn using the function msavisr from the package seqvisr (version 0.2.7) (https://rdrr.io/github/vragh/seqvisr/, accessed on 23 December 2023).

The RT-LAMP_ONNV_ primer design was based on the genomic sequence of the ONNV Gulu strain (GenBank accession number M20303.1), the same strain used in the infection dynamics experiments. The RT-LAMP_ONNV_ primer set, containing six primers—the outer primers (F3 and B3; or forward and reverse primers, respectively), the inner primers (FIP and BIP), and the loop primers (LB and LF)—was designed using the PrimerExplorer V5 program (Eiken Chemical Co., Ltd., Tokyo, Japan; https://primerexplorer.jp/e/, accessed on 13 February 2024). Whenever possible (see Section 3 for details), the limit of a ΔG value of ≤−4 kcal/mol (the ideal stability of the primer ends) was not exceeded. All DNA primers were purchased from STAB VIDA (Caparica, Portugal) with HPLC purification quality.

### 2.5. Optimization of the RT-LAMP_ONNV_ Assay

Before establishing the RT-LAMP_ONNV_ assay, several reaction conditions were optimized, including different concentrations of MgSO_4_ (2 mM, 4 mM, 6 mM, 8 mM), various reaction temperatures (60 °C, 64 °C, 66 °C, 68 °C, 70 °C), different reaction times (10, 20, and 30 min), different outer/inner/loop primer ratios (1:8:2, 1:4:2, 1:4:1, 1:8:0, 1:4:0), and the effect of adding betaine to the reaction mixture. After optimization, unless stated otherwise, RT-LAMP reactions were performed with a final concentration of 0.2 µM for the F3 and B3 primers, 1.6 µM for the FIP and BIP primers, and 0.4 µM for the loop primers. The reaction mixtures also included 1.4 mM dNTP (1.4 mM each), 8 U of *Bst* 3.0 polymerase (NEB), 0.5 M of betaine, 4 mM of MgSO_4_, 1× of Isothermal Amplification Buffer II (20 mM Tris-HCl, 10 mM [NH_4_]_2_SO_4_, 150 mM KCl, 2 mM MgSO_4_, 0.1% Tween^®^ 20, pH 8.8 at 25 °C; commercially available at NEB), and 1 μL of the RNA extract, in a total reaction volume of 25 μL. The amplification reactions were set at 64 °C for 30 min, unless specified otherwise. The readout of a positive or negative result was determined by the emission of fluorescence in the tubes after the addition of 1 µL of DMSO (Sigma-Aldrich, St. Louis, MO, USA) and 1 µL of SYBR Green I (NZYtech, Lisbon, Portugal). Additionally, the validation of positive or negative amplifications in RT-LAMP_ONNV_ reactions was conducted by loading a 5 µL aliquot of the RT-LAMP products in a 2% agarose gel stained with ethidium bromide, which was then electrophoresed for 40 min at 110 V. Following electrophoresis, the gel was examined under a UV-transilluminator (ChemiDoc™ Bio-Rad, Hercules, CA, USA). Successful amplifications were identified by the presence of ladder-type DNA bands.

### 2.6. Limit of Detection of RT-LAMP_ONNV_

Ten μL of the original ONNV viral extract (from BEI Resources, with a titer of 1.2 × 10^7^ pfu/mL) was initially diluted in 90 μL of PBS, and this dilution was then subjected to heating at 95 °C for 5 min, bypassing the RNA extraction step. The limit of detection of the RT-LAMP_ONNV_ assay was determined using 10-fold serial dilutions of this heated sample. For each corresponding reaction, 1 µL of each 10-fold serial dilution was used to perform the RT-LAMP_ONNV_ assay.

### 2.7. Specificity of RT-LAMP_ONNV_

To evaluate the specificity of the RT-LAMP_ONNV_ assay, RNA extracted from virus stocks, produced by infecting Vero E6 cells with ONNV (see Section 2.2), was used as the template. Additionally, three other alphaviruses—namely, CHIKV (strain S-27; laboratory stock), SINV (strain EfAr339; laboratory stock), and MAYV (strain TRVL-4675, laboratory stock recently prepared after obtaining the virus through BEI Resources-NIAID-NIH)—all belonging to the Semliki Forest serological complex (all stored at −80 °C after replication on VeroE6 cells) were used for viral RNA extraction and included in the assay’s specificity evaluation. Each extracted RNA sample was diluted 1:1000 in nuclease-free water, and 1 µL of each RNA extract was used as the template for RT-LAMP_ONNV_. ONNV was used as a positive control, and water was used as a negative control (NTC).

## 3. Results

### 3.1. Phylogenetic Analysis of Alphavirus

Using a comprehensive dataset of 58 complete alphavirus genomes, we employed multiple sequence alignment followed by phylogenetic tree construction using the maximum likelihood method to place ONNV in the context of its attributed genus, including its placement on the branch indicating the antigenic Semliki Forrest serocomplex (Figure S2). In a general analysis, three major clades were identified in the obtained phylogenetic tree: one corresponding to the so-called Old-World alphaviruses, another to New-World alphaviruses, and a third one including aquatic alphaviruses, with the latter corresponding to the tree outgroup. As expected, ONNV was positioned within the Old-World alphavirus clade, where its phylogenetically closeness to CHIKV, to which it shares common ancestry, became evident. In a more detailed analysis, we observed that ONNV is also phylogenetically close to MAYV, both positioned within the Old-World alphavirus clade, although MAYV is, indeed, a New-World alphavirus (Figure S2). Considering this result, when designing primers and assessing the specificity of RT-LAMP_ONNV_, other alphaviruses circulating in the same regions as ONNV, such as CHIKV, needed to be considered along with MAYV.

### 3.2. RT-LAMP_ONNV_ Primer Design

The primers used in the course of this work were designed for the Gulu strain of ONNV (Table 1).

**Figure 1:**
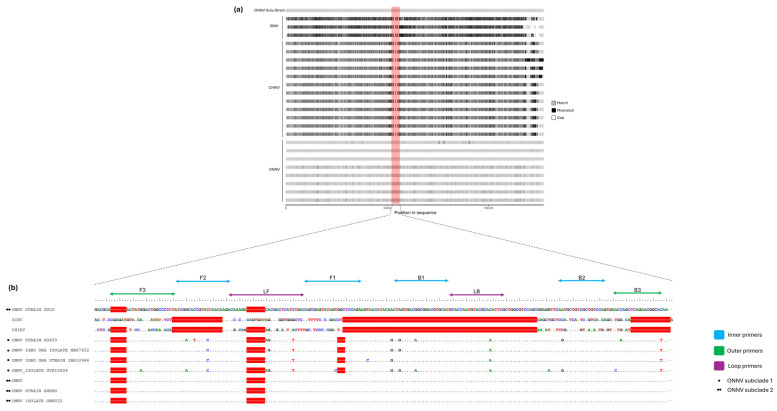
Multiple alignment overview of the design of primers. (**a**) Multiple alignment scheme of ONNV, SINV, and CHIKV genomes, showing each position of the genomes, whether they are a match, mismatch, or gap relative to the reference genome ONNV Gulu strain. The region of interest for LAMP primer design is shown in red. (**b**) Detailed view from Bioedit of the region of interest. Bars with arrows represent each primer design for RT-LAMP_ONNV_. The gap between genomes relative to the reference genome ONNV Gulu strain is shown in red. Each subclade of ONNV (reference Figure S2) is represented by subclade 1 (●) and subclade 2 (●●).

A multiple sequence alignment (MSA) of nucleotides was performed with all available complete genomes of ONNV, three genomes of SINV, and a representative genome of each lineage of CHIKV (except for the West Africa lineage, where all available genomes were included since it is the region where ONNV also circulates). The MSA revealed (Figure 1a) that a potential region of interest (ROI) was suitable for primer design, as it had two key characteristics potentially contributing to the intended high specificity of RT-LAMP_ONNV_: it was a highly polymorphic region, and it appeared to be specific only for ONNV genomes (Figure 1b).

This region, which stands out in the alignments of ONNV/CHIKV/SINV genomes as part of a gap, does not seem to be an artifact of the alignment process, but rather results from the larger size of the ONNV genome in this region. This observation was confirmed using an MSA of amino acid sequences that showed that the encoded polyprotein of CHIKV and SINV is, indeed, smaller than that of ONNV, which makes this ROI specific to the ONNV genome (Figure 1).

After identifying the ROI, the LAMP primers were designed using Primer Explorer V5 software. The goal was to define not only the four necessary primers (F3 and B3—outer primers; FIP and BIP—inner primers) for the intended RT-LAMP, but also to design loop primers (LF and LB) that simultaneously enhance the reaction efficiency and decrease the reaction time. The first attempt, using the default conditions defined in the Primer Explorer V5 software, resulted in the design of the F3, B3, FIP, BIP oligonucleotides, yet it was impossible to find matching loop primers. Alternatively, we decreased the minimum length of each primer to 18 base pairs and decreased the minimum Tm value to 63 °C for F1c/B1c (which constitutes part of FIP and BIP). This adjustment allowed the design of the F3, B3, FIP, and BIP primers. To design the loop primers to match the corresponding four LAMP oligonucleotides, the conditions of the program were further adjusted by decreasing the GC rate to a minimum of 30%, setting the length of the primers to a minimum of 10 base pairs, and finally, decreasing the Tm value to a minimum of 58 °C. Even though one of the key factors in designing RT-LAMP_ONNV_ primers is the stability at the end of the primers, where the free energy should be −4 kcal/mol or less, two of them (F3 and B1c) had free energy values slightly higher than −4 kcal/mol. This compromise was accepted due to the high specificity of the targeted ONNV genome region by these primers.

### 3.3. Optimization of Assay Conditions for RT-LAMP_ONNV_

To investigate the optimal conditions for the RT-LAMP_ONNV_ assay, six parameters were tentatively optimized: the MgSO_4_ concentration, reaction temperature, outer/inner/loop primer ratio, reaction time, enzyme quantity, and use of additives (betaine in this case). The RT-LAMP_ONNV_ reaction was performed under basal conditions: 2 mM of MgSO_4_ (the concentration present in the buffer), 60 °C for 30 min, a 1:8:2 primer ratio, and 8 U *Bst* 3.0, with no betaine added.

**Figure 2:**
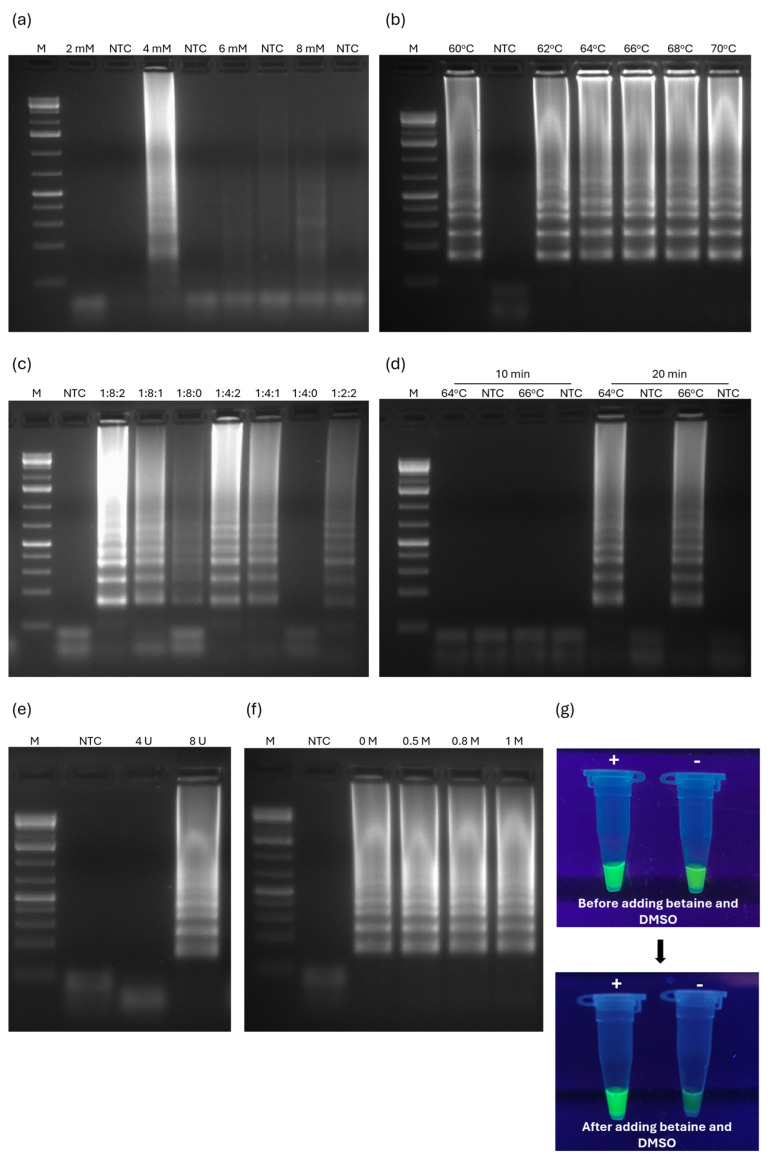
Optimization of RT-LAMP_ONNV_ conditions. (**a**) Effect of varying concentrations of MgSO_4_ on the RT-LAMP_ONNV_ reaction; (**b**) different reaction temperatures to determine the optimal temperature for the RT-LAMP_ONNV_ reaction; (**c**) agarose gel analysis of the impact of different ratios of outer/inner/loop primers on the RT-LAMP_ONNV_ reaction; (**d**) agarose gel analysis for comparison of the results of the RT-LAMP_ONNV_ reaction at two temperatures (64 °C and 66 °C) over different reaction times; (**e**) agarose gel analysis of the effect of varying concentrations of *Bst* 3.0 polymerase on the RT-LAMP_ONNV_ reaction; (**f**) agarose gel analysis of the impact of different concentrations of betaine on the RT-LAMP_ONNV_ reaction; (**g**) effect of adding betaine and DMSO to reaction result visualization with SYBR Green I. M: Molecular marker—GeneRuler 1 kb Plus DNA Ladder (Invitrogen, Carlsbad, CA, USA); NTC: Negative control; +/−: Positive/negative RT-LAMP_ONNV_ result.

We began by optimizing the MgSO_4_ concentration, as *Bst* 3.0 activity is highly dependent on it. Four different MgSO_4_ concentrations, ranging from 2 mM to 8 mM, were tested. As shown in Figure 2a, no reaction occurred with the minimum concentration of 2 mM. Although positive reactions were observed at higher concentrations (Figure 2a), the most intense ladder-type DNA bands appeared at 4 mM. Consequently, all subsequent RT-LAMP_ONNV_ reactions were performed with 4 mM MgSO_4_.

Next, to determine the optimal reaction temperature, the RT-LAMP assay was performed using a temperature gradient ranging between 60 and 70 °C (Figure 2b). The ONNV genome was detected at every reaction temperature, but the band intensity was slightly greater at 64 and 66 °C (Figure 2b).

To find the best outer/inner/loop primer ratio for RT-LAMP_ONNV_ performance, seven ratios were tested, covering the presence/absence of loop primers and variations of the inner primer concentration (Figure 2c). The ratio used as the basal condition (1:8:2) yielded the best results, with strong amplification intensity (Figure 2c). Notably, the absence of loop primers affected the reaction efficiency, resulting in weaker (1:8:0) or even negative (1:4:0) amplification results (Figure 2c). However, even with half the amount of inner primers (1:4:2), the inclusion of loop primers in the reaction mixtures enhanced amplification, yielding positive results with strong band intensity (Figure 2c). This indicated that the optimal primer ratio for the RT-LAMP_ONNV_ assay was 1:8:2.

On the other hand, to reduce the reaction time, RT-LAMP_ONNV_ was performed at 64 and 66 °C with reaction times of 10 and 20 min (Figure 2d). The amplification of ONNV sequences was not observed if the reaction time lasted only 10 min, but 20 min was sufficient to produce unambiguously positive amplification results at both temperatures (Figure 2d). Therefore, subsequent reactions were conducted at 64 °C for 20 min.

In addition, to determine if enzyme usage could be reduced while still producing positive results, RT-LAMP_ONNV_ was performed using half the recommended 8 U of *Bst* 3.0 enzyme and with all the conditions optimized until this point (Figure 2e). No positive amplification results were observed on the agarose gel (Figure 2e), indicating that 8 U of *Bst* 3.0 was, indeed, necessary.

Finally, since adding betaine to a LAMP reaction can increase its specificity [36], we tested the effect of adding different betaine concentrations (0.5 M, 0.8 M, and 1 M) on the performance of RT-LAMP_ONNV_. As shown in Figure 2f, there were no significant visual differences between the DNA amplified in the presence or absence of betaine, regardless of its concentration.

Although successful LAMP amplification can be confirmed by inspecting the characteristic dumbbell-shaped DNA in agarose gels, colorimetric or turbidimetric detection provides quicker results and reduces the risk of cross-contamination from aerosol formation during tube handling. Therefore, we decided to use SYBR Green I to distinguish between a positive and a negative amplification result. However, the visualization using SYBR Green I did not yield the best results due to the background noise produced by self-primer dimers (Figure 2g), which were visible in the agarose gels. To overcome this issue, we decided to still use it in the reaction mixtures, even though it did not significantly impact RT-LAMP_ONNV_ performance (see above). Adding 0.5 M of betaine enhanced the visual difference between positive and negative results with SYBR Green I, improving assay sensitivity and specificity (Figure 2g). We also reduced the primer concentration by lowering the primer ratio to 1:4:1, which decreased the likelihood of self-primer dimers. Furthermore, adding 1 µL of DMSO at the end of the reaction, which destabilizes nonspecific primer interactions, improved the visualization and differentiation between positive and negative results (Figure 2g), making it possible to apply the assay for on-field testing.

After optimization, the RT-LAMP_ONNV_ conditions were defined as 4 mM of MgSO_4_, 64 °C, a 1:4:1 outer/inner/loop primer ratio, a 20 min reaction time, 0.5 M of betaine, and 8 U of *Bst* 3.0.

### 3.4. Establishing Limit of Detection Thresholds and Evaluation of RT-LAMP_ONNV_ Assay Specificity

**Figure 3:**
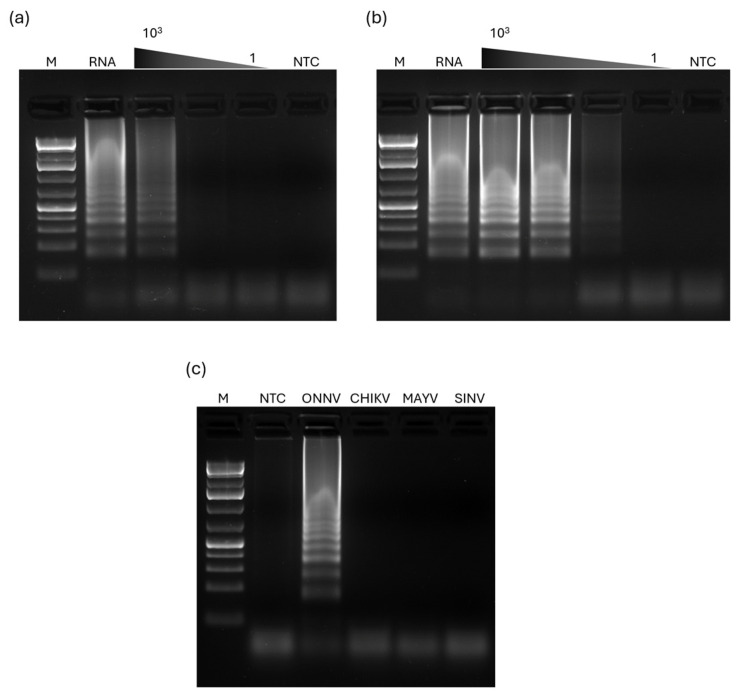
Comparison of the limit of detection of RT-LAMP_ONNV_ between two different reaction times and specificity of RT-LAMP_ONNV_. (**a**) Limit of detection for a 20 min reaction time. (**b**) Limit of detection for a 30 min reaction time. (**c**) Agarose gel electrophoresis analysis of the specificity of the assay to detect ONNV, when performed with RNA of CHIKV, MAYV, and SINV. M: Molecular marker—GeneRuler 1 kb Plus DNA Ladder (Invitrogen). NTC: Negative control. RNA: ONNV RNA extract.

The limit of detection of the RT-LAMP_ONNV_ assay was determined using 10-fold serial dilutions of the original viral stock from BEI Resources, which was diluted in PBS and heat-treated (see Materials and Methods, Section 2.6 for details) (Figure 3a,b). Starting with a 10^−2^ dilution (meaning 10^5^ pfu/reaction) of the heat-treated viral stock, we prepared additional serial dilutions to assess the detection limit of the RT-LAMP_ONNV_ assay, enabling the potential detection of as few as 1 pfu of ONNV per reaction.

**Figure 4:**
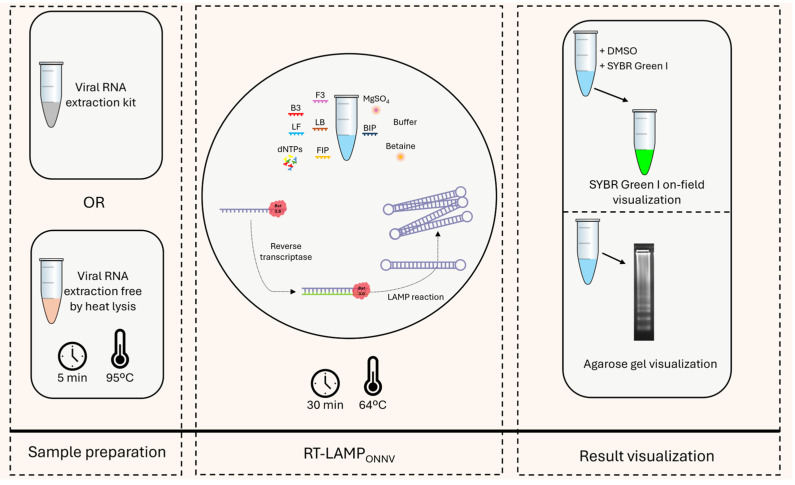
Schematic overview of RT-LAMP_ONNV_.

As the reaction time had been optimized to 20 min (see above), initially, the definition of the RT-LAMP_ONNV_ detection limit was carried out accordingly (Figure 3a), resulting in a limit of detection of 10^3^ pfu/reaction. However, to explore the impact of extending the reaction time by 10 min—from 20 to 30 min—on the assay detection limit, we tested this change despite its minimal effect on amplification efficiency with purified RNA, and as shown (Figure 3b), this extension allowed the detection of ONNV at concentrations as low as 10 pfu/reaction, establishing this as the RT-LAMP_ONNV_ assay’s limit of detection. Thus, the final conditions for performing this RT-LAMP_ONNV_ were as described in Section 3.3, except for the reaction time, which was increased to 30 min, improving the limit of detection (Figure 4).

To evaluate the specificity of the RT-LAMP_ONNV_ assay, we compared it with three other viruses, CHIKV, MAYV, and SINV (Figure 3c).

As shown, no positive amplification was visible in the agarose gel for CHIKV, MAYV, and SINV (Figure 3c). In contrast, ONNV showed a clear and intense positive signal (Figure 3c). This indicates that the RT-LAMP_ONNV_ assay is specific to ONNV and can effectively distinguish it from other closely related viruses, particularly the phylogenetically close CHIKV.

## 4. Discussion and Conclusions

In recent years, the spread of vectors transmitting human pathogenic viruses such as CHIKV, ZIKV, and DENV has expanded in temperate regions like Europe [37,38,39], where these viruses can be either potentially fatal or have severe health consequences. This trend is largely driven by climate change, which creates optimal conditions for vector reproduction, leading to their establishment [40]. Along with globalization and shortened travel times, the risk of viral outbreaks has risen as infected individuals can rapidly transport viruses across continents, potentially introducing them to new regions as patients.

The increasing presence of these vectors has highlighted the urgent need for effective diagnostic tools. These tools are crucial not only in new regions where viruses spread but also in their areas of origin, where their circulation often goes undetected. Many infections caused by arboviruses are either asymptomatic or present with no specific symptoms when clinically manifested, making diagnosis challenging. Furthermore, in malaria-endemic regions, viral infections are often either misdiagnosed as malaria or mistaken for bacterial infections [41]. Even when a viral infection is implicated, laboratory confirmation is rare and typically relies on serologic methods. These methods often suffer from cross-reactivity, especially in areas where antigenically related viruses co-circulate, with confirmation requiring expensive and technically demanding approaches (such as PRNT), which must be carried out under adequate biocontainment conditions. On the other hand, molecular identification typically requires expensive equipment and highly trained personnel. As a result, ONNV, an alphavirus virus closely related to CHIKV, often suffers from misidentification due to serological cross-reactivity and the lack of rapid, relatively less technically demanding molecular alternatives.

Isothermal amplification techniques, such as LAMP (or RT-LAMP if the target of detection is RNA rather than DNA, as in the case of the genomes of human arboviruses), have emerged as promising solutions due to their simplicity and effectiveness. Over the years, numerous virus-specific RT-LAMP techniques have appeared in the literature [27,42,43,44,45,46], none of which require expensive equipment or intensive technical training, making them particularly suitable for virus detection in low-resource regions. To address this, we developed a rapid, highly sensitive, and specific RT-LAMP assay for ONNV detection. The assay, named RT-LAMP_ONNV_, was optimized to detect approximately 10 pfu/reaction in minimally processed samples, showing high specificity to ONNV with no cross-reactivity to other alphaviruses, particularly CHIKV.

The design of LAMP primers is critical and more challenging than for PCR, requiring four to six primers for a small region of interest [47]. Based on the features of a region of interest, where the genome of ONNV differs sufficiently from that of other alphaviruses such as SINV or the closely related CHIKV, and in the performance of the PrimerExplorer, we designed the needed primers for a LAMP-type amplification, including the reacting efficiency-boosting loop primers, specific to ONNV. The primers were designed specifically for the only available strain of ONNV (the Gulu strain). However, as shown in Figure S1, ONNV genomes are phylogenetically classified into two subclades, indicating that the additional optimization of primers may be needed (through degeneration) to cover the entire genetic variability of ONNV and ensure the detection of all circulating strains/variants with high sensitivity. Indeed, Figure 1b shows polymorphisms between the two subclades but their impact on RT-LAMP_ONNV_ sensitivity could not be assessed.

We utilized the *Bst* 3.0 polymerase for its strand displacement and reverse transcriptase activities, which minimize tube manipulations and reduce contamination risks, a significant issue given the assay’s high sensitivity [36]. To mitigate possible cross-contamination, we compartmentalized the workflow, conducting different steps in separate areas. Indeed, cross-contamination may be a vulnerability of the assay, if applied to on-field diagnosis, given the power of the technique. RT-LAMP_ONNV_ also simplified the RNA extraction process. Despite ONNV’s lipid envelope [48] and structured capsid [49], we used heat-mediated lysis on untreated viral suspensions to release RNA, eliminating the RNA extraction step, and allowing a more accurate assessment of the RT-LAMP_ONNV_ limit of detection.

The RT-LAMP_ONNV_ performance characteristics indicated its potential to significantly enhance ONNV detection and support rapid response efforts during outbreaks or for routine viral surveillance. This is particularly valuable in regions where ONNV co-circulates with related alphaviruses and in resource-limited settings impacted by climate change. For point-of-care diagnosis, where the clear visualization of a positive result is crucial for on-field application, we selected a simple method of positive/negative visualization using SYBR Green I. Although we encountered issues with self-primer dimer formation, a common problem with this technique [50], the use of betaine and DMSO helped resolve this, allowing us to employ the end-point visual detection of LAMP products for on-field diagnosis.

Comparison with other ONNV detection methods is challenging because ONNV remains an under-studied arbovirus, with limited available information. However, some methods do exist. For instance, a multiplex detection assay using antibodies [51] offers high specificity, although there are no data available regarding its cost, reaction time, sensitivity, or limit of detection (LOD). Another available detection method is RT-PCR [52], which has a similar LOD to our RT-LAMP_ONNV_. However, the cost associated with RT-PCR, including equipment, reagents, and specialized personnel, makes it impractical for field diagnostics in countries where ONNV is endemic. In conclusion, RT-LAMP_ONNV_ presented here offers a promising tool for the specific, rapid, and sensitive detection of ONNV. Further optimization and field testing could expand its applicability, contributing to better management and control of viral outbreaks in diverse settings.

## Figures and Tables

**Table 1:** Designed primers for the detection of ONNV through RT-LAMP_ONNV_. 5′ΔG and 3′ΔG correspond to Gibbs free energy of the 5′ and 3′ end, respectively. This parameter should be less than −4 kcal/mol, considering the ideal stability of the primer ends.

Primer	5′ΔG	3′ΔG	Sequence (5′–3′)
F3	−3.12	−4.27	AACTATGGACTGGCCCTCTA
B3	−7.08	−5.00	TGCCGTTCTGAGCTGGTT
FIP	-	-	TCTGGAGCCACTATACTCGCTG-TCGGCACCGTATCAACAAG
BIP	-	-	TAGTGACGGCGGACGTGCAT-CTGGACAGCGACAGCATT
LF	−4.15	−4.16	TCAGATGAGTCGTGTCTTTGT
LB	−6.41	−4.84	GCACCAATGTACGCACATTCG

## Data Availability

The original contributions presented in the study are included in the article/Supplementary Material; further inquiries can be directed to the corresponding author.

## Supplementary Materials

The following supporting information can be downloaded at: https://www.mdpi.com/article/10.3390/pathogens13100892/s1, Figure S1: Cytopathic effects in Vero E6 cells inoculated with ONNV Gulu strain. [I] Mock-infected and infected Vero E6 cells with ONNV Gulu strain for 96 h. [II] Detailed observation of the cytopathic effects in Vero E6 cells produced by the ONNV infection, including cell rounding and detachment (shown with red arrows); Figure S2: Phylogenetic analysis. (a) Maximum likelihood phylogenetic analysis of Alphaviruses. The position of ONNV is highlighted in bold. Each color represents the classification of the virus: Old-World alphaviruses (green), New-World alphaviruses (red), and aquatic alphaviruses (blue), with the aquatic alphaviruses serving as the outgroup. (b) Maximum likelihood phylogenetic analysis of ONNV and 5 representative genomes of each CHIKV lineage. Each color represents a CHIKV lineage: Asia lineage (dark blue), IOL lineage (orange), ESCA lineage (green), Americas lineage (blue), and West Africa lineage (red). For the clade of ONNV, each subclade is represented by subclade 1 (●) and subclade 2 (●●). At branches, * indicates those supported by aLRT or a bootstrap value >75% (of 1000 data resamplings), whereas ** indicates both aLRT/bootstrap support.
