# Supplementary figures and images for: High-Sensitivity RT-LAMP for Molecular Detection of O’nyong-nyong (Alphavirus onyong)
Source: Pathogens. 2024 Oct 11;13(10):892. doi: 10.3390/pathogens13100892 (PMC11510077; doi:10.3390/pathogens13100892)

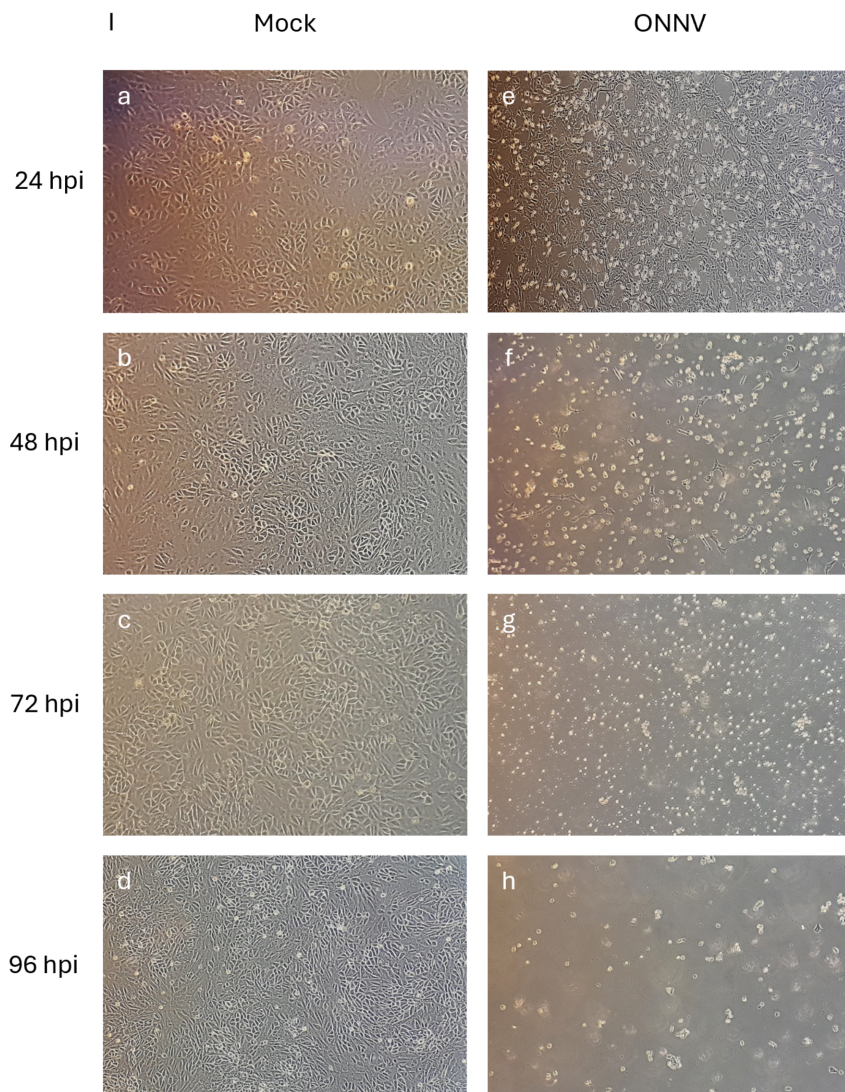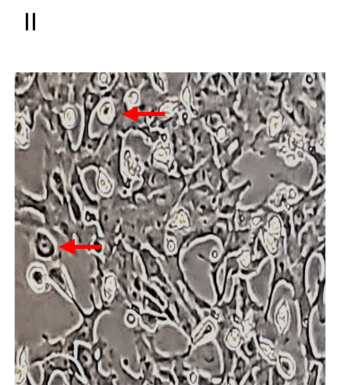

Supplement: Supplementary file 1 [file pathogens-13-00892-s001.zip › FigureS1_400_dpi.pdf]

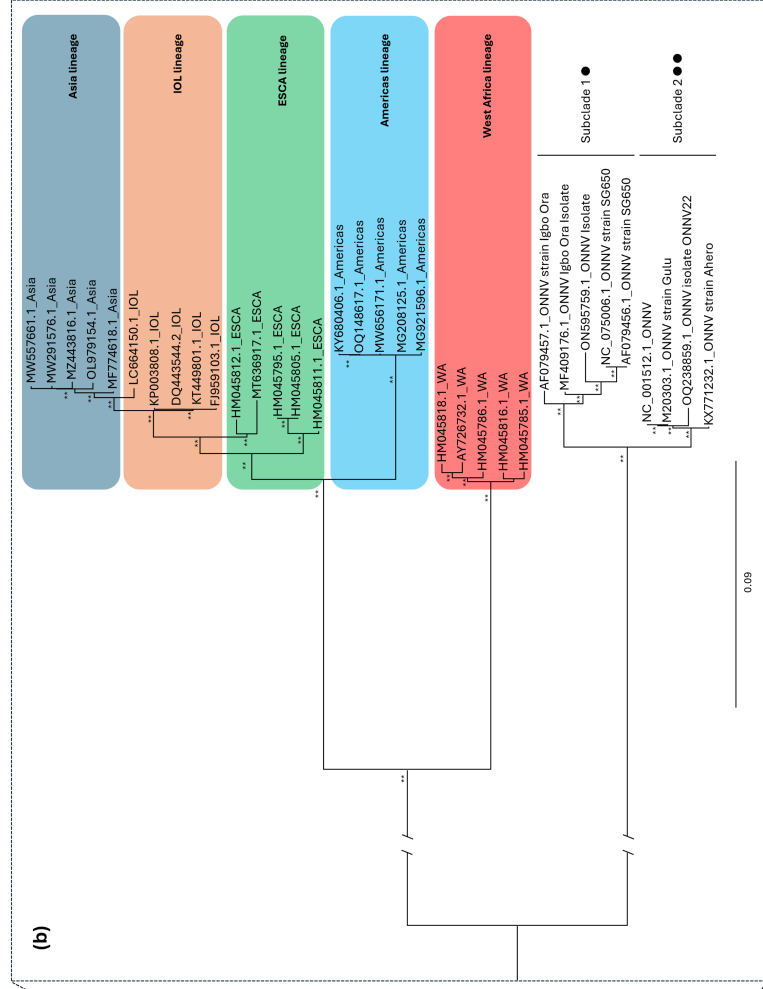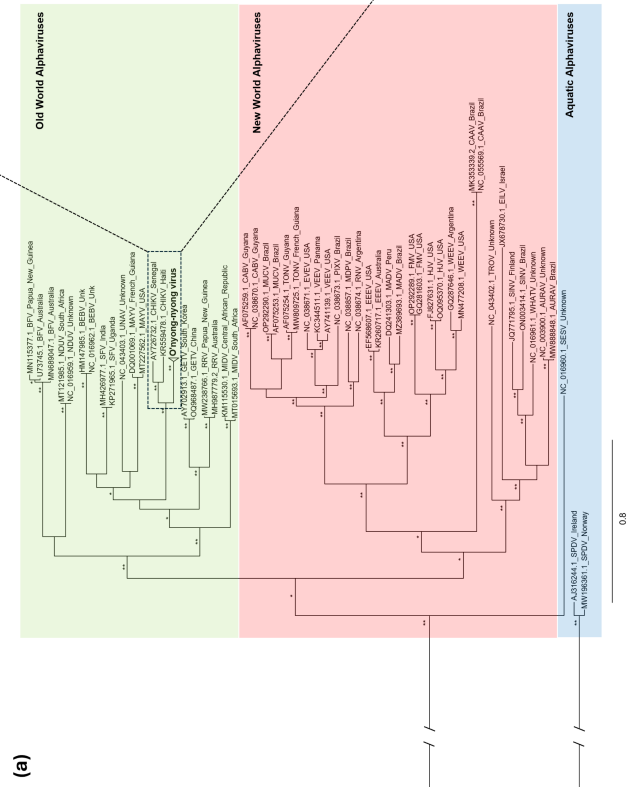

Supplement: Supplementary file 1 [file pathogens-13-00892-s001.zip › FigureS2_400_dpi.pdf]
